# Blocking Aδ- and C-fiber neural transmission by sub-kilohertz peripheral nerve stimulation

**DOI:** 10.3389/fnins.2024.1404903

**Published:** 2024-07-15

**Authors:** Shaopeng Zhang, Longtu Chen, Sajjad Rigi Ladez, Ahmet Seferge, Jia Liu, Bin Feng

**Affiliations:** Department of Biomedical Engineering, University of Connecticut, Storrs, CT, United States

**Keywords:** neuromodulation, peripheral nerve stimulation, sub-kilohertz, nerve block, action potential

## Abstract

**Introduction:**

We recently showed that sub-kilohertz electrical stimulation of the afferent somata in the dorsal root ganglia (DRG) reversibly blocks afferent transmission. Here, we further investigated whether similar conduction block can be achieved by stimulating the nerve trunk with electrical peripheral nerve stimulation (ePNS).

**Methods:**

We explored the mechanisms and parameters of conduction block by ePNS via ex vivo single-fiber recordings from two somatic (sciatic and saphenous) and one autonomic (vagal) nerves harvested from mice. Action potentials were evoked on one end of the nerve and recorded on the other end from teased nerve filaments, i.e., single-fiber recordings. ePNS was delivered in the middle of the nerve trunk using a glass suction electrode at frequencies of 5, 10, 50, 100, 500, and 1000 Hz.

**Results:**

Suprathreshold ePNS reversibly blocks axonal neural transmission of both thinly myelinated Aδ-fiber axons and unmyelinated C-fiber axons. ePNS leads to a progressive decrease in conduction velocity (CV) until transmission blockage, suggesting activity-dependent conduction slowing. The blocking efficiency is dependent on the axonal conduction velocity, with Aδ-fibers efficiently blocked by 50–1000 Hz stimulation and C-fibers blocked by 10–50 Hz. The corresponding NEURON simulation of action potential transmission indicates that the disrupted transmembrane sodium and potassium concentration gradients underly the transmission block by the ePNS.

**Discussion:**

The current study provides direct evidence of reversible Aδ- and C-fiber transmission blockage by low-frequency (<100 Hz) electrical stimulation of the nerve trunk, a previously overlooked mechanism that can be harnessed to enhance the therapeutic effect of ePNS in treating neurological disorders.

## 1 Introduction

Sensory afferent neurons are arguably the longest cells in the body; each consists of a soma at the dorsal root ganglia (DRG), a peripheral axon projecting to the end organ, a central axon terminating in the dorsal horn of spinal cord, and a stem axon forming a T-junction with the peripheral and central axons (Lawson, 2005; Abraira and Ginty, 2013). Blocking the afferent neural transmission to the spinal cord has been extensively applied in the clinic for managing various sensory-related disorders, especially many types of chronic pain arising from sensitized afferent input from peripheral tissues (Epstein and Palmieri, 2012; Verrills et al., 2016). In particular, patients suffering from nociceptive pain, postherpetic neuralgia, peripheral neuropathy, musculoskeletal pain, and visceral pain report significant pain relief from treatments that block afferent drives from the spinal nerves (Eldabe et al., 2018; Huygen et al., 2020; Berger et al., 2021; Kim et al., 2021; Chapman et al., 2023). Migraines and headaches can also be managed by blocking afferent drives from the trigeminal ganglia pathway (Messlinger and Russo, 2019). In addition, blocking vagal afferent drive shows therapeutic efficacy in treating obesity and neuropathic cough (Roslin and Kurian, 2001; Canning et al., 2006).

Pharmacological approaches are implemented to block peripheral afferent drives in the clinics. Peripherally restricted kappa-opioid receptor agonists show analgesic efficacy in chronic visceral pain and neuropathic pain in animal models and clinical trials (Albert-Vartanian et al., 2016). Eluxadoline, a peripherally restricted small molecule drug with dual agonist/antagonist action on opioid receptor subtypes, has been approved by the FDA for treating visceral pain associated with irritable bowel syndrome (Lembo et al., 2016). Another dual-acting delta/kappa opioid receptor agonist CAV1001, has shown promising pain attenuating effects in the treatment of arthritis pain, neuropathic pain, and bone cancer pain (Hartrick et al., 2020). Also, cannabinoid receptor agonists activating only peripheral CB1 receptors (CB1Rs) have been reported to effectively alleviate multiple chronic pain conditions with limited side effects (Zhang et al., 2018). Peripheral nerve block (PNB) by local anesthetic injection effectively relieves acute pain (Shah et al., 2018). With advancement in catheter delivery of anesthetics and chemical wrapping to extend the anesthetic’s release, PNB has also been applied to treat chronic pain conditions like migraine headache, chronic pelvic pain syndrome, and chronic Achilles’ tendinopathy (Koo and O'Brien, 2011; Shauly et al., 2019; Chimenti et al., 2020).

Peripheral sensory tissue ablation is an irreversible procedure used to block the afferent drives. Electrical radiofrequency (RF) ablation removes target nerves and tissues by thermal damage, which is effective in suppressing chronic hip pain, chronic discogenic back pain, chronic thoracic and abdominal pain, and knee osteoarthritis pain (Davis et al., 2018; Kumar et al., 2019; Kapural and Deering, 2020; Kim et al., 2020; Singh et al., 2021). Chemical neurolysis uses substances like ethanol or phenol to irreversibly damage peripheral tissues, e.g., celiac plexus for managing chronic upper abdominal pain (Cornman-Homonoff et al., 2017; Sachdev and Gress, 2018) and sacral dorsal rami for sacroiliac joint pain (Nouer Frederico et al., 2021). In addition, cryoablation resorts to extreme low temperature (−60 to −100°C) to ablate peripheral tissues for treating pain (Yasin et al., 2020).

In addition to the pharmacological and tissue ablation approaches, electrical stimulation of peripheral nerves by either anodic direct current or charge-balanced alternating stimulation at kilohertz range can reversibly block afferent neural transmission, as thoroughly reviewed recently (Kilgore and Bhadra, 2014; Patel and Butera, 2018). Anodal block leads to imbalanced electro-chemical reactions at the electrode-tissue interface and is usually reserved as a research tool to selectively block myelinated axons (Sassen and Zimmermann, 1973; Whitwam and Kidd, 1975; Petruska et al., 1998). Charge-balanced kilohertz stimulation reversibly blocks peripheral nerves with rapid onset (within seconds) and modest carry-over effects (seconds to minutes after terminating the stimulus), and has demonstrated efficacy in clinical applications (Patel and Butera, 2018).

Kilohertz frequency stimulation typically ranges from 5 to 100 kHz and has been investigated for its ability to block different types of nerve fibers. Myelinated fast-conductive A-fibers are often blocked at lower amplitudes compared to slow-conductive unmyelinated C-fibers (Patel and Butera, 2018). Studies have shown that higher amplitudes are required to block unmyelinated C-fibers effectively, which may result in non-selective blocking when targeting mixed nerve populations (Patel and Butera, 2015).

Various animal models have been used to study the effects of kilohertz frequency (KHF) block. For instance, Bhadra et al. demonstrated the reversible block of the sciatic nerve in rats using high frequency alternating current with frequencies from 10 to 30 kHz at amplitudes of between 2 and 10 V. Their study showed that high frequency alternating current can induce a reversible nerve conduction block without causing long-term damage to the nerve fibers (Bhadra and Kilgore, 2005). Recent research by Pelot et al. extended these findings by quantifying the effects of KHF on the rat vagus nerve, revealing that block thresholds increase monotonically with frequency for both fast and slow nerve fibers, and that neural conduction can take tens of seconds to recover following certain KHF application. This suggests that while KHF can effectively block nerve activity, the recovery time may vary depending on the duration and amplitude of the applied signal (Pelot and Grill, 2020). Despite these promising results, there are important limitations to consider, kilohertz stimulation does not seem to offer very high selectivity in blocking unmyelinated C-fibers over myelinated A-fiber (Patel and Butera, 2015). This limited selectivity is a critical consideration in the development and application of electrical blocking techniques for therapeutic use. As reported in our recent study, sub-kilohertz electrical stimulation of the afferent somata in the DRG offers frequency-dependent transmission block of C-fiber and Aδ-fiber afferents in an *ex vivo* preparation for single-fiber recordings from colorectal afferents (Chen et al., 2022). The optimal blocking frequency (OBF) for C-fiber afferents is at 20–50 Hz, while the OBF for Aδ-fiber afferents is at 50–100 Hz. The underlying mechanism of transmission block is likely through activity-dependent conduction slowing, and thus requires supra-threshold stimulation (Chen et al., 2022). However, it remains undetermined whether electrical peripheral nerve stimulation (ePNS) at sub-kilohertz range could also achieve frequency-dependent afferent transmission block. In the current study, we harvested three different peripheral nerves from mice, i.e., the sciatic, saphenous, and vagal nerves, and conducted *ex vivo* single-fiber recordings to study the afferent transmission block by ePNS of the nerve trunk. We also performed complementary computational simulations in the NEURON environment to provide a mechanistic interpretation of the axonal transmission block by peripheral nerve stimulation.

## 2 Methods

All experiments were reviewed and approved by the University of Connecticut Institutional Animal Care and Use Committee. All the mice used in the following experiments were housed in pathogen-free facilities which are Public Health Service assured and American Association for Accreditation of Laboratory Animal Care accredited following the Guide for the Care and Use of Laboratory Animals Eighth Edition. Mice resided in individual ventilated caging systems in polycarbonate cages (Animal Care System M.I.C.E.) and were provided with contact bedding (Envigo T7990 B.G. Irradiated Teklad Sani-Chips). Mice were fed *ad lib* with either 2,918 Irradiated Teklad Global 18% Rodent Diet or 7,904 Irradiated S2335 Mouse Breeder Diet supplied by Envigo and supplied with reverse osmosis water chlorinated to 2 ppm using a water bottle. Nestlets and huts were supplied for enrichment. Rodent housing temperature was set for 73.5°F with a range from 70 to 77°F. Humidity was set at 50% with a range of 35 to 65%. Mice were housed with a maximum of 5 animals per cage. All animals were housed on a 12: 12 light–dark cycle. Animals were observed daily by the animal care services staff. Cages were changed every 2 weeks.

### 2.1 *Ex vivo* experimental setup for single-fiber recordings from peripheral nerves

From adult C57BL/6 mice of both sexes (aged 10–16 weeks, weighing 25–35 g), we harvested two spinal nerves: the sciatic and saphenous nerves containing axons from DRG neurons, and the vagal nerve containing axons from the nodose ganglia neurons, following a surgical procedure previously reported (Chen et al., 2022). Briefly, mice were anesthetized with 2% isoflurane inhalation and euthanized by transcardiac perfusion with oxygenated Krebs solution (in mM: 117.9 NaCl, 4.7 KCl, 25 NaHCO_3_, 1.3 NaH_2_PO_4_, 1.2 MgSO_4_, 2.5 CaCl_2_, and 11.1 D-glucose at room temperature) from the left ventricle to the right atrium through the circulatory system. The perfused carcass was then immediately transferred to a dissection chamber circulated with oxygenated ice-cold Krebs solution for nerve harvesting. The sciatic nerve, approximately 35–40 mm in length, was meticulously harvested from its proximal projection at the L4 spinal cord to its distal projection at the tibial nerve in the heel. The saphenous nerve, approximately 30–35 mm long, was harvested from its proximal projection at the L4 spinal cord and traced along the femoral nerve down to its terminal branch at the medial side of the foot. The vagal nerve, approximately 20–30 mm long, was harvested from its proximal projection at the nodose ganglia around the neck and continuously dissected along its path to its distal branches at the diaphragm separating the thoracic and abdominal chambers.

**Figure 1:**
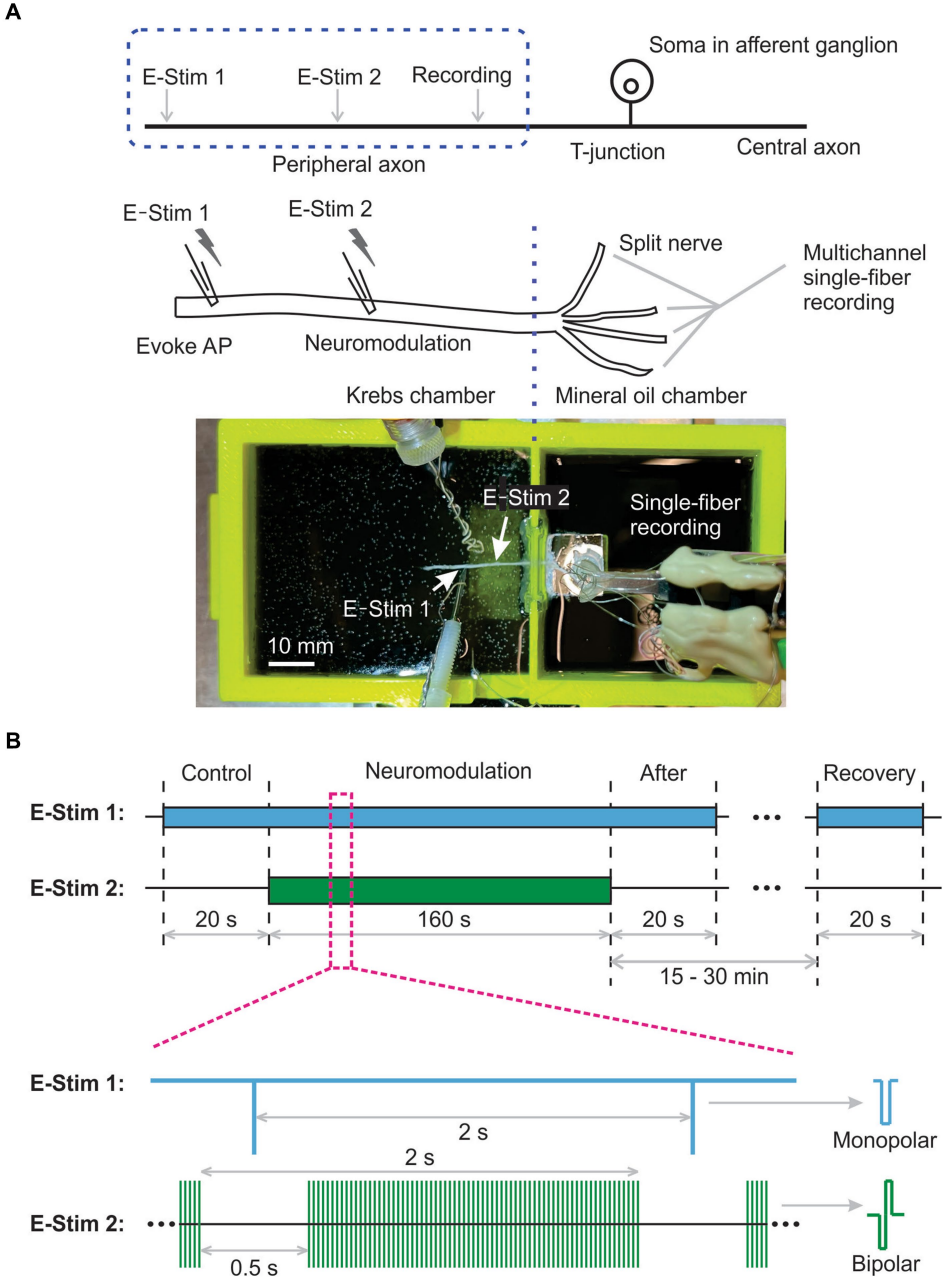
Schematic of multichannel single-fiber recordings to assess neural transmission block by electrical peripheral nerve stimulation (ePNS). **(A)** The diagram and photo showing the *ex vivo* single-fiber recordings from a peripheral nerve harvested from mice. **(B)** The synchronized stimulation protocol to study the effect of ePNS on axonal neural transmission.

As shown in Figure 1A, harvested nerves were then transferred to a custom-built two-compartment chamber consisting of a tissue compartment and a recording compartment (Chen et al., 2017a). The proximal end of the targeted nerve was pinned down in the tissue compartment circulated with oxygenated Krebs solution at 28–30°C. The ~5 mm distal end of the targeted nerve was gently pulled over and laid onto a mirror in the recording compartment filled with mineral oil (Fisher Scientific, East Greenwich, RI) to enhance the signal-to-noise ratio of the recording. Then, the distal end of the nerve (recording chamber side) was split into fine filaments (~10 μm thickness) to achieve optimal single-fiber recordings from individual afferent/efferent axons (Chen et al., 2022). A customized 5-channel electrode array was utilized to interface with split nerve filaments (Chen et al., 2017b). Single-unit action potentials (APs) from all five electrodes were recorded simultaneously, digitized at 20 kHz and band-pass filtered (200–3,000 Hz) using an Intan RHD USB interface board. The multichannel recording signals were also monitored by a data acquisition system (1401plus, CED, Cambridge, UK) and stored onto a PC using Spike2 software (v7.1, CED, Cambridge, UK).

### 2.2 Neuromodulation protocols to assess axonal transmission block by peripheral nerve stimulation

Neural transmission initiation occurred at the distal end of the harvested nerve, indicated by “E-Stim 1” in Figure 1A. Neuromodulation to block transmission was evaluated by peripheral nerve stimulation at the site marked “E-Stim 2” in Figure 1A. Both stimulations utilized custom-built liquid suction electrodes, created by pulling quartz glass capillaries in a micropipette puller (P-97, Sutter Instrument, Novato, CA) to form a tip approximately 30% smaller than the nerve diameter, i.e., ~Φ400 μm for the sciatic and saphenous nerves and ~ Φ200 μm for the vagal nerve. These electrodes, filled with Krebs solution, formed a loose seal with the epineurium surface via gentle negative pressure (−60 to −30 mmHg), with care taken to clean surrounding connective tissue to aid seal formation.

APs were evoked at “E-Stim 1” every 2 s using constant current stimulation (cathodic, monophasic, 0.5 Hz, 0.2 ms pulse width, >5 times threshold amplitude) from a programmable stimulus isolator (A-M Systems 4100, Carlsborg, WA). Neuromodulation via ePNS (charge-balanced bipolar stimulation, cathodic first) was delivered to site “E-Stim 2,” about 5 to 10 mm from “E-Stim 1,” using another programmable stimulus isolator (A-M Systems 4100, Carlsborg, WA).

The stimulus thresholds of current amplitudes to evoke APs from the same axon were determined at both the “E-Stim 1” and “E-Stim 2” sites, established by 0.5 Hz stimulation evoking 4 to 6 APs per 10 stimuli. The stimulus intensity at “E-Stim 1” exceeded 5 times the threshold amplitude (0.1–0.5 mA) to ensure robust AP generation. Neuromodulation intensities at “E-Stim 2” were set as either suprathreshold or subthreshold, corresponding to ~150% and ~80% of the threshold current amplitude, respectively.

To characterize the instantaneous neuromodulation effect of ePNS, we implemented a synchronized stimulation protocol at both “E-Stim 1” and “E-Stim 2,” as depicted in Figure 1B. The pulse frequency for evoking APs at “E-Stim 1” and the train frequency of neuromodulation at “E-Stim 2” were both set at 0.5 Hz. The pulse frequency of neuromodulation at “E-Stim 2” was set at 5, 10, 50, 100, 500, or 1,000 Hz. The train duration was set at 1.5 s followed by a 0.5 s intertrain interval. AP transmission was continuously monitored by the “E-Stim 1” during the 0.5 s interval between the neuromodulation trains, which was sufficiently long to prevent interference from the stimulus artifacts generated by the neuromodulation at “E-Stim 2.”

The study protocol comprised a 20-s control stimulation at “E-Stim 1” only, followed by a 160-s synchronized stimulation at both “E-Stim 1” and “E-Stim 2” to assess neuromodulation. This was succeeded by a 20-s stimulation at “E-Stim 1” immediately post-neuromodulation, and another 20-s stimulation 15 to 30 min later to assess AP transmission recovery from neuromodulation.

### 2.3 Computational simulation of axonal transmission block by peripheral nerve stimulation

**Figure 2:**
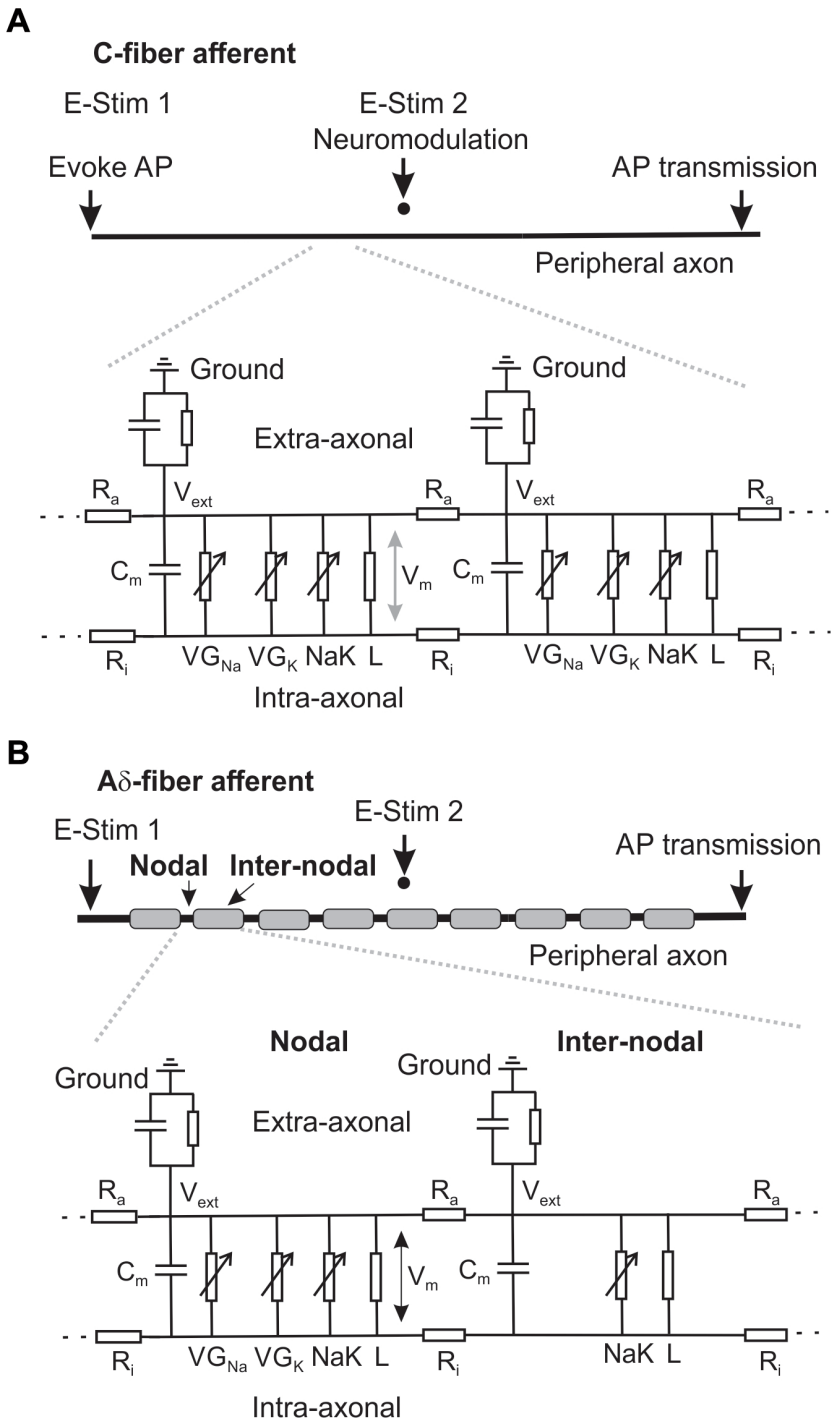
Schematic of the neural membrane models that simulate the AP transmission in an unmyelinated C-fiber **(A)** and a thinly myelinated Aδ-fiber axon **(B)**.

Two double-cable models were built in NEURON for simulating a thinly myelinated Aδ-fiber afferent and an unmyelinated C-fiber afferent, respectively. As shown in Figure 2A, the geometry of the C-fiber model consists of a single component: a fiber with a length of 10 mm and a diameter of 1.2 μm. The fiber is discretized into 1,000 segments so that each segment is 10 μm in length. This discretization enables the representation of spatial variations in membrane properties and facilitates numerical simulations of neural activity along the fiber. The Aδ-fiber model as illustrated in Figure 2B includes the nodal and the internodal regions. Each nodal region is represented by a single segment of 5 μm in length and 2.4 μm in diameter. Each 30 μm-long internodal region (2.4 μm in diameter) is discretized into 10 segments. The peripheral fiber of 5 mm long consists of 143 pairs of nodal and inter-nodal regions. The segment lengths for both Aδ- and C-fiber models are less than 1/10 of their respective space constants λ (Plonsey and Barr, 2007). To ensure the accuracy of the simulations, additional convergence studies were conducted using the C-fiber model with a larger discretization of 20 μm segments. The results obtained from the 20 μm discretization showed no significant differences compared to those obtained using the 10 μm discretization.

The assessment of ePNS Neuromodulation on axonal AP transmission is illustrated in Figures 2A,B, showing the AP initiation from the peripheral end at 0.4 Hz by intra-axonal current injection (0.5 nA, 0.75 ms duration) at “E-Stim 1” and transmission to the central end. To simulate extracellular stimulation, a point current source is placed 20 μm above the middle point of the fiber in both the Aδ and C-fiber models as indicated by “E-Stim 2.”

#### 2.3.1 Modeling the extracellular stimulation

To be consistent with the current *ex vivo* experimental study of axonal transmission block, we modeled the axons in a homogeneous conductive saline solution with a conductivity of 1.45 S/m (Sauerheber and Heinz, 2015). The ePNS was modeled as extracellular stimulation from a point source positioned 20 μm above the middle of the axon in both Aδ- and C-fiber models, which generates electrical potentials at each segment of the model to excite the axons. To simulate non-zero extracellular potential, an “extracellular” point process was inserted into each segment of the axonal model in the NEURON simulation environment. The extracellular voltage distributions across the Aδ- and C-fiber models generate transmembrane currents, which drive action potential generation during extracellular electrical stimulation at site “E-stim 2.”

#### 2.3.2 Passive electrophysiological properties and initial conditions

The initial ionic concentrations for the C-fiber and Aδ-fiber models are: 140 mM for extracellular sodium ([Na^+^]_o_), 4.5 mM for intracellular sodium ([Na^+^]_i_), 5 mM for extracellular potassium ([K^+^]_o_), and 130 mM for intracellular potassium concentrations ([K^+^]_i_) (Feng et al., 2015). The initial resting membrane potential was set at −65 mV. Simulations were executed at a temperature of 30°C, consistent with the current *ex vivo* experimental condition. The C-fiber model is composed of homogeneous segments, whereas the Aδ-fiber model features alternating nodal and internodal regions to simulate the saltatory conduction of action potentials through the nodes of Ranvier. The internodal regions are insulated by a thick myelin sheath and lack voltage-gated sodium and potassium channels. In contrast, the nodal region, which includes the nodes of Ranvier, paranodal, and juxtaparanodal regions, has a high concentration of voltage-gated sodium and potassium channels. For unmyelinated axons and the nodal region, the membrane capacitance (Cm) was set at 1 μF/cm^2^. At the internodal regions, the membrane capacitance was set to 0.01 μF/cm^2^ to reflect myelination. The intracellular resistivity Ri was set at 123 Ω∙cm throughout. The leak membrane conductance was set at 0.001 S/cm^2^ with a reversal potential of −60 mV. The intra-axonal diffusion coefficients for both Na^+^ and K^+^ were set as 0.6 μm^2^/ms (Feng et al., 2015).

#### 2.3.3 Ion channels and pump

The C-fiber model incorporates four voltage-gated sodium channel conductances (NaV1.6, NaV1.7, NaV1.8, NaV1.9) and three voltage-gated potassium channel conductances (fast-inactivating A-type K_A_, slowly inactivating A-type K_D_, and sustained K_S_). NaV1.6 and NaV1.7 are modeled with Markov-type formulations to depict their contrasting gating characteristics, i.e., rapid vs. gradual repriming and incomplete vs. complete inactivation. The other ion channels are modeled by Hodgkin-Huxley-type formulations adopted from our prior study (Feng et al., 2015). The ionic pump Na, K-ATPase (NaKA) is also included, producing an outward current based on a 3: 2 Na^+^/K^+^ transport ratio. Intracellular Na^+^ and K^+^ concentrations are modeled to change constantly from transmembrane ionic fluxes via ion channels, NaKA and leak conductance as well as axial intra-axonal diffusion.

In the Aδ-fiber model, similar compositions of ion channels are included at the nodal region except for the absence of NaV1.8, which are typically present in unmyelinated DRG neurons (Renganathan et al., 2001). At the internodal regions of the Aδ-fiber, transmembrane conductance consists of NaKA and the leak channel only, with no other ion channels. The maximum ion channel conductance or pump current in both models are listed in Table 1.

**Table 1:** Maximum ion channel conductance or pump current.

	A-fiber (internodal)	A-fiber (nodal)	C-fiber
Na_V_1.6, pS/μm^2^	0	1,400	1,400
Na_V_1.7, pS/μm^2^	0	2,000	2,500
Na_V_1.8, pS/μm^2^	0	0	3,000
Na_V_1.9, pS/μm^2^	0	1	1
K_A_, pS/μm^2^	0	600	700
K_D_, pS/μm^2^	0	400	600
K_S_, pS/μm^2^	0	300	300
Na^+^, K^+^-ATPase, pA/μm^2^	0.001	0.001	0.25
Leak conductance, pS/μm^2^	10	10	10

#### 2.3.4 Data analysis and statistics

Action potentials recorded by single-fiber recordings were processed off-line using customized MATLAB program. The detection thresholds for individual action potentials were set as four times the root mean square (RMS) amplitude of background noise recorded 10 ms before the stimulation. Conduction delays were measured as the time between the onset of stimulus artifacts and the onset of recorded action potentials. The conduction velocity (CV) was computed from the conduction delay and the distance between the “E-Stim 1” and the recording site. Data are presented as means ± standard error (S.E.). One-way ANOVA was performed as appropriate using SigmaStat v4.0 (Systat Software, San Jose, CA). Differences were considered significant when *p* < 0.05.

## 3 Results

### 3.1 Recording APs from the same axon by stimulation at both “E-stim 1” and “E-stim 2” sites

**Figure 3:**
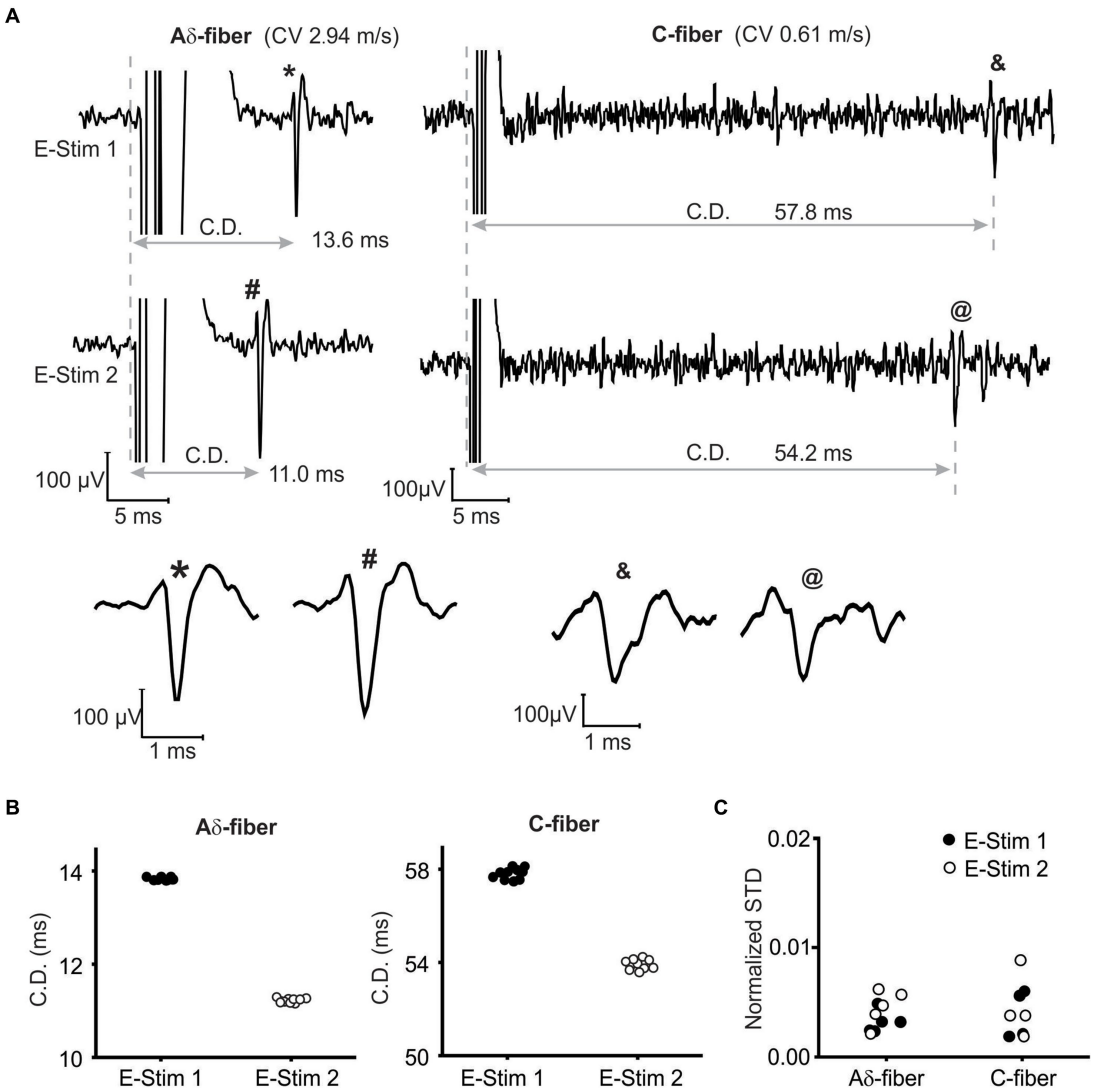
Single-fiber recordings of APs from the same axon when stimulated at both the initiation (E-Stim 1) and neuromodulation (E-Stim 2) sites. **(A)** Representative recordings from an Aδ-fiber and a C-fiber axon. The extended view indicated comparable AP waveform from both stimulation sites. **(B)** The conduction delays (CD) of APs in **(A)** from 10 consecutive stimulation (0.5 Hz) at both sites. **(C)** Normalized standard deviations (STD) of 10 consecutive CD as calculated by dividing the STD with the mean CD.

To assess suprathreshold ePNS on AP transmission, we first validated that APs evoked by the stimulation at both the initiation (E-Stim 1) and neuromodulation sites (E-Stim 2) can be reliably recorded from the distal end of the nerve via single-fiber recordings. Displayed in Figure 3A are typical single-fiber recordings of APs from an Aδ- and a C-fiber axon when stimulated at either E-stim 1 or E-stim 2 site. APs evoked at both sites at 0.5 Hz showed no appreciable activity-dependent changes in the conduction delays (CD) as evidenced by the recordings from 10 consecutive stimulation (Figure 3B). As summarized in Figure 3C, the standard deviation (STD) of the 10 consecutive CD is less than 1% in 5 Aδ-fiber axons and 4 C-fiber axons tested (normalized STD < 0.01).

### 3.2 Reversible transmission block of peripheral axons by sub-kilohertz ePNS

**Figure 4:**
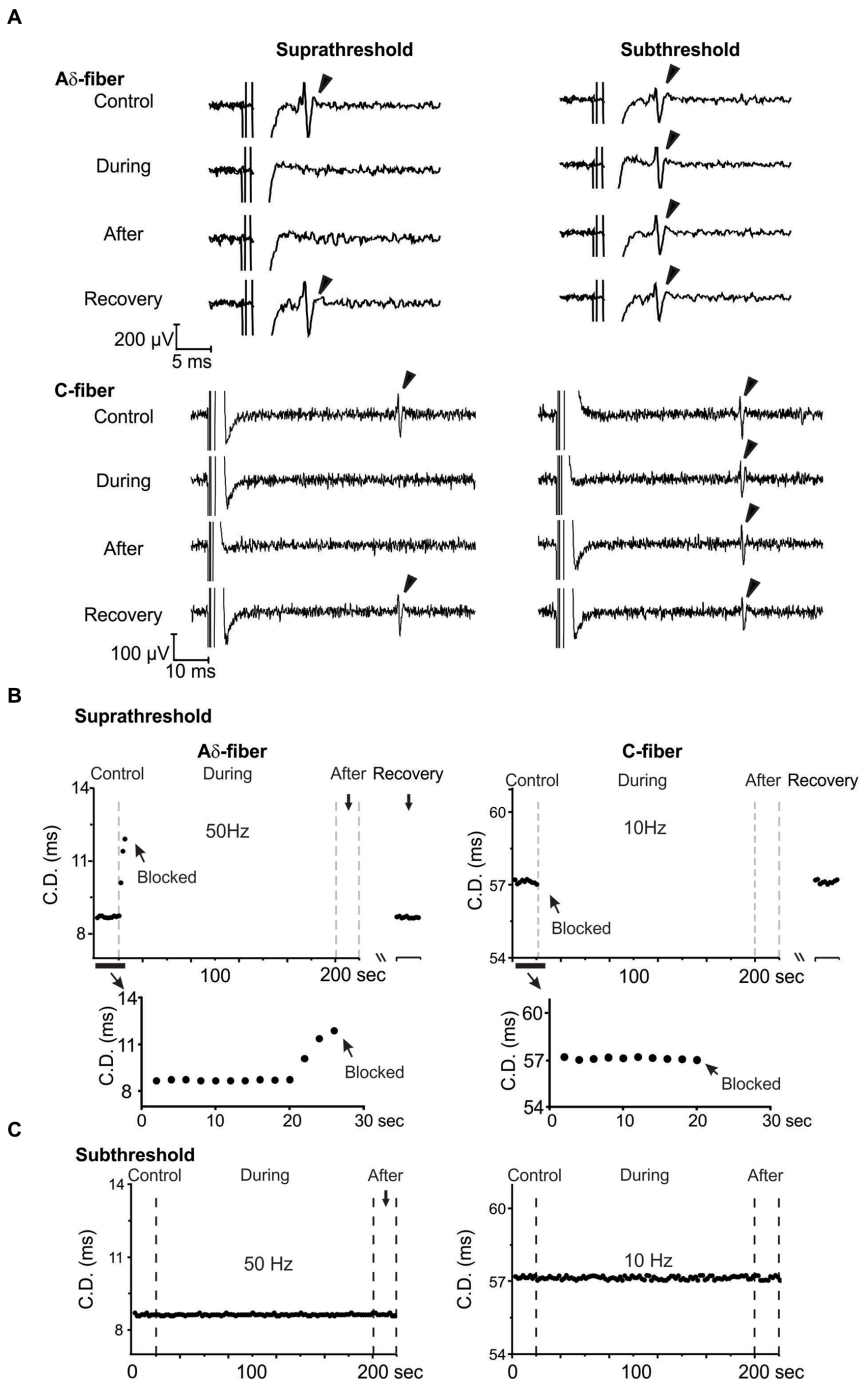
Reversible transmission block of Aδ- and C-fiber axons by suprathreshold ePNS at 50 and 10 Hz, respectively. **(A)** Representative single-fiber recordings from Aδ- and C-fiber axons before, during, immediately after, and 15–30 min after the neuromodulation delivered at “E-Stim 2.” Displayed in **(B,C)** are representative CD recorded once every 2 s during the suprathreshold and subthreshold ePNS, respectively.

As shown in the study protocol in Figure 1B, single-fiber recordings of APs evoked from “E-Stim 1” were conducted before (control), during, immediately after, and 15–30 min after ePNS neuromodulation (recovery) delivered at “E-Stim 2.” The threshold current amplitude required to activate the same axon differs between “E-Stim 1” and “E-Stim 2” due to variations in the stimulus configurations of the suction electrodes at the two sites, including differences in seal resistance and the distance between the axon and the electrode tip. The threshold current amplitudes for activating Aδ-fiber axons range from 0.12 to 1.5 mA, while the thresholds for activating C-fiber axons from 0.8 to 3 mA. Displayed in Figure 4A are typical recordings of APs from an Aδ- and a C-type axon showing the neuromodulatory effect on the AP transmission by both subthreshold (80%) and suprathreshold (150%) stimulations. Action potentials were evoked at “E-Stim 1” by a 0.5 Hz stimulation. Displayed in Figure 4B are typical CD recorded from an Aδ- and a C-fiber axon throughout the course of the suprathreshold stimulation protocol. The extended views showing the onset of ePNS at “E-stim 2” revealed a progressive increase in CD in the Aδ-fiber axon till transmission block and an instantaneous transmission block in the C-fiber axon. The CD from the same axons were subjected to the subthreshold stimulation protocol and displayed in Figure 4C, which showed no significant changes throughout the stimulation.

### 3.3 Frequency-dependent axonal transmission block by ePNS

**Figure 5:**
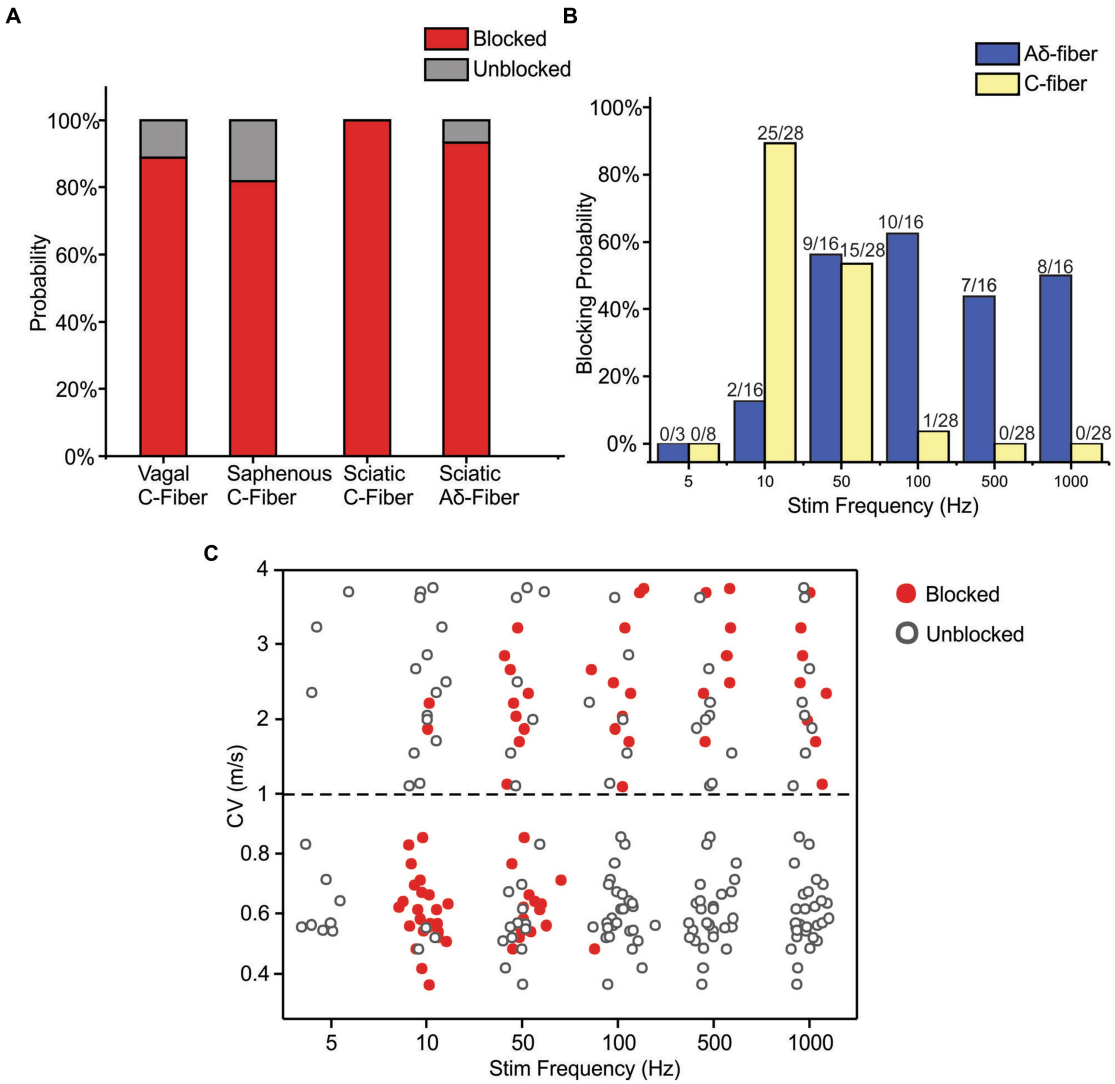
Frequency-dependent axonal transmission block by suprathreshold ePNS assessed at six different frequencies from 5 to 1,000 Hz. **(A)** The blocking probability of Aδ- and C-fiber axons by at least one of the six stimulus frequencies in vagal, saphenous, and sciatic nerves. **(B)** The blocking probability of Aδ- and C-fiber axons by ePNS at six different stimulus frequencies. **(C)** The CV of axons that are blocked (solid dots) or unblocked (open dots) by ePNS at different frequencies.

The neuromodulatory effects of sub-kilohertz ePNS were studied in 25 mice focusing on two different types of axons, i.e., Aδ- fiber axons with CV from 1 to 4 m/s (*N* = 16) and C-fiber axons with CV less than 1 m/s (*N* = 28), consistent with the range of CV reported in mouse Aδ- and C-fiber axons (Koltzenburg et al., 1997; Lawson, 2002). All the 16 Aδ-fibers were recorded from the sciatic nerve and the 28 C-fibers were from the sciatic (*N* = 8), saphenous (*N* = 11), and vagal (*N* = 9) nerves. Each axon was assessed with five suprathreshold ePNS protocols that deliver stimuli frequencies at 10, 50, 100, 500, and 1,000 Hz, respectively. In 3 out of 16 Aδ-fiber and 8 out of 28 C-fiber axons, ePNS was also assessed at a lower frequency of 5 Hz. Axonal transmission block was defined as 10 consecutive transmission failures of APs evoked from “E-Stim 1” at 0.5 Hz. Summarized in Figure 5A are the blocking probabilities of Aδ- and C-fiber by at least one of the six frequencies of ePNS protocols (5 to 1,000 Hz). Most, if not all C-fibers were blocked by ePNS (in 88.9% vagal, 81.8% saphenous, and 100% sciatic C-fiber axons). There is no significant difference in the blocking probability of C fibers across different nerve types (Chi-square test, *p* > 0.05). Thus, we pooled the C-fiber data from different peripheral nerves together for subsequent analyses.

The frequency-dependent transmission block by ePNS was summarized in Figure 5B, showing that C-fiber axons are optimally blocked by 10 and 50 Hz stimulation with blocking probabilities over 53.6% while Aδ-fiber axons are optimally blocked by 50–1,000 Hz stimulation with blocking probabilities over 50%. No transmission block was observed with 5 Hz ePNS in either Aδ- or C-fiber axons. In particular, ePNS of 10 Hz selectively blocked C-fibers over Aδ-fibers (Fisher’s exact test, *p* < 0.001), while ePNS frequency of 100 Hz and above selectively blocked Aδ-fibers over C-fibers (*p* < 0.001 for 100, 500, and 1,000 Hz). At 50 Hz, ePNS blocks comparable proportions of Aδ-and C-fibers (*p* = 1.0).

The CV of the blocked versus unblocked axons were summarized in Figure 5C, indicating that the blocking effect is not only dependent on the stimulus frequency but also related to the CV of the individual axon. Stimulus frequencies of 10 Hz efficiently blocked 77% of afferents with CV from 0.3 to 2.2 m/s, but did not block a single axon with CV over 2.2 m/s. The frequency of 50 Hz stimulation is more effective at blocking afferents with CV from 0.5 to 3.3 m/s, including both the C- and Aδ-fiber axons. Stimulation at 100, 500, and 1,000 Hz by ePNS selectively blocked Aδ-fibers with almost no blocking effect on C-fibers. Stimulation at 5 Hz did not block any tested axons.

### 3.4 Increase in conduction delay following peripheral nerve stimulation

**Figure 6:**
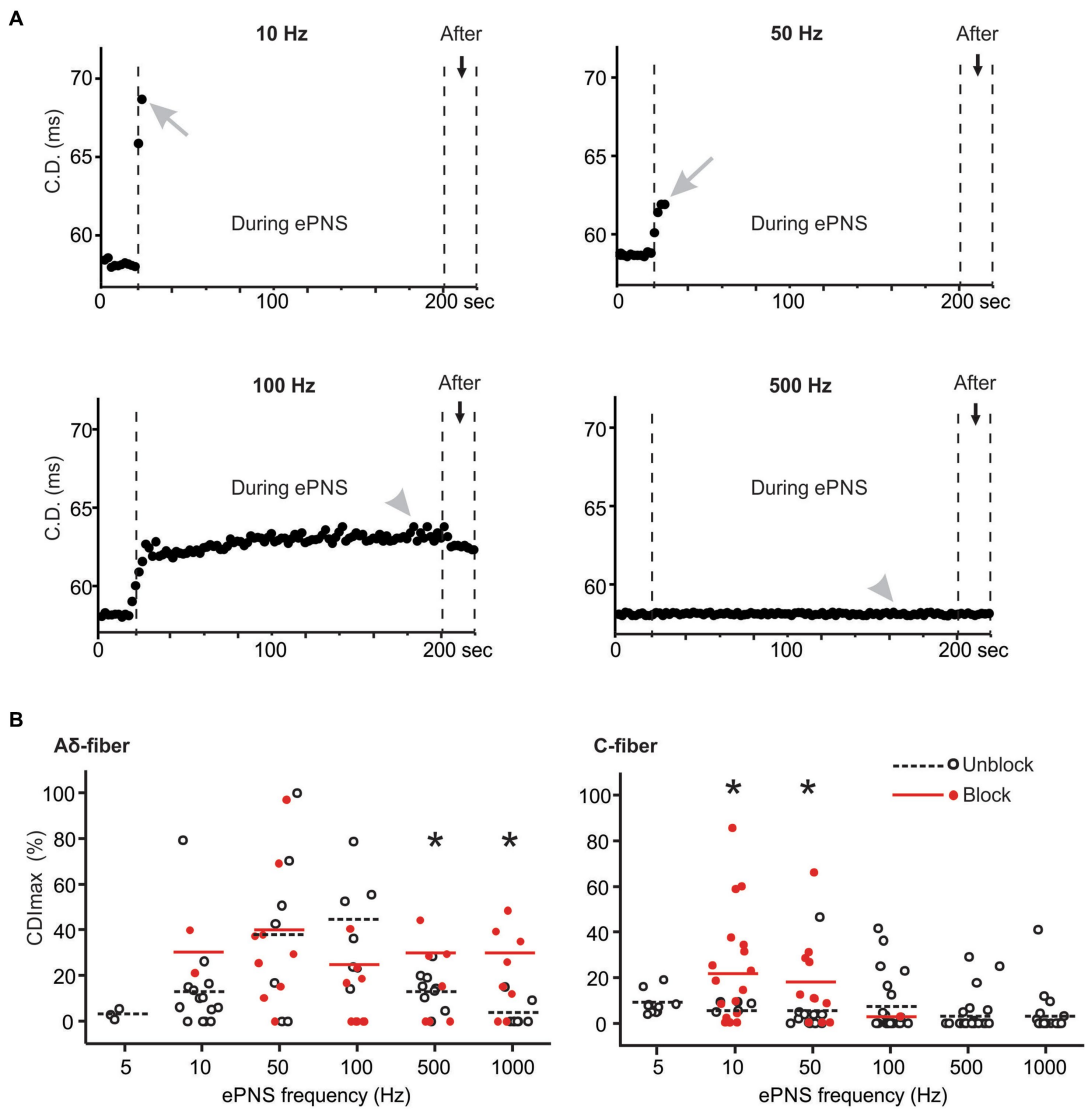
The conduction delay increase (CDI) in blocked and unblocked axons. **(A)** representative CD recorded from one C-fiber axon undergoing ePNS at four different frequencies. **(B)** Summary of maximum CDI (CDI_max_) in blocked and unblocked axons. * indicates *p* < 0.05 between blocked and unblocked CDI_max_.

The synchronized stimulation protocol as described in Figure 1B enables the monitoring of CD once every 2 s during the ePNS protocol. The change in conduction delay was determined in 148 ePNS protocols in C-fiber axons and 83 protocols in Aδ-fiber axons. Aδ-type fibers have CV between 1 and 4 m/s and C-type fiber less than 1 m/s (Chen et al., 2022). Displayed in Figure 6A are CD from one representative axon subjected to four different ePNS frequencies; transmission block was achieved at 10 and 50 Hz stimulation but not at the higher frequencies of 100 and 500 Hz. The conduction delay increase (CDI) was normalized as percentage increase to pre-stimulus CD, and the maximum CDI (CDI_max_) was determined during the ePNS protocol. In the blocked axon, the CDI_max_ usually occurred right before the conduction block as indicated by gray arrows in Figure 6A, while in the unblocked axon the CDI_max_ was often at the plateau region toward the end of the neuromodulation (gray arrowhead). In 12.1% of the ePNS protocols (17/148 C-fibers, 11/83 Aδ-fibers), the transmission block was achieved right after the first 1.5 s of stimulation (e.g., the C-fiber axon in Figure 4B), precluding the measurement of CDI_max_. Those recordings were excluded in the analysis in Figure 6B, which summarizes the CDI_max_ in blocked (red solid dots) and unblocked axons (black open dots) at five ePNS frequencies (10–1,000 Hz). Stimulation at 5 Hz resulted in minimal CDI and those data were excluded from subsequent analyses. At their preferred blocking frequencies, the CDI_max_ is significantly higher in blocked axons compared to unblocked ones for both Aδ-fibers (*t*-test, *p* = 0.049 for 500 Hz, *p* = 0.005 for 1,000 Hz) and C-fibers (*p* = 0.003 for 10 Hz, *p* = 0.030 for 50 Hz). However, at midrange frequency (50 and 100 Hz), there was no observed difference in CDI_max_ between blocked and unblocked Aδ-fiber axons. Comparisons were not conducted at frequencies with low blocking probabilities (<20%) for either Aδ-fiber or C-fibers.

### 3.5 Computational simulations reveal that disrupted transmembrane sodium and potassium concentrations underly the transmission block by ePNS

In our investigation, we compared the simulated action potential (AP) waveforms generated by our computational models representing Aδ- and C-fiber afferents with the empirical data obtained via whole-cell patch-clamp experiments as detailed by Zheng et al. (2019). The half-maximum widths of the APs in our models were found to be 2.4 ms for C-fibers and 0.4 ms for Aδ-fibers, consistent with the reported experimental findings of 2–2.36 ms for mouse C-fiber afferents (i.e., C-LTMRs) and 0.3–0.4 ms for A-fiber afferents (Aδ-LTMRs, Aβ-LTMRs, and proprioceptors) (Zheng et al., 2019). Likewise, our model simulations yielded peak amplitudes of 46 mV for C-fiber and 40 mV for Aδ-fiber afferents, consistent with the experimental observations (Zheng et al., 2019). Conduction velocities (CV) were determined based upon the simulated conduction delay from one end of the axon to the other end and the geometric length, showing CV of 0.53 m/s for the C-fiber model and 1.6 m/s for the Aδ-fiber model.

**Figure 7:**
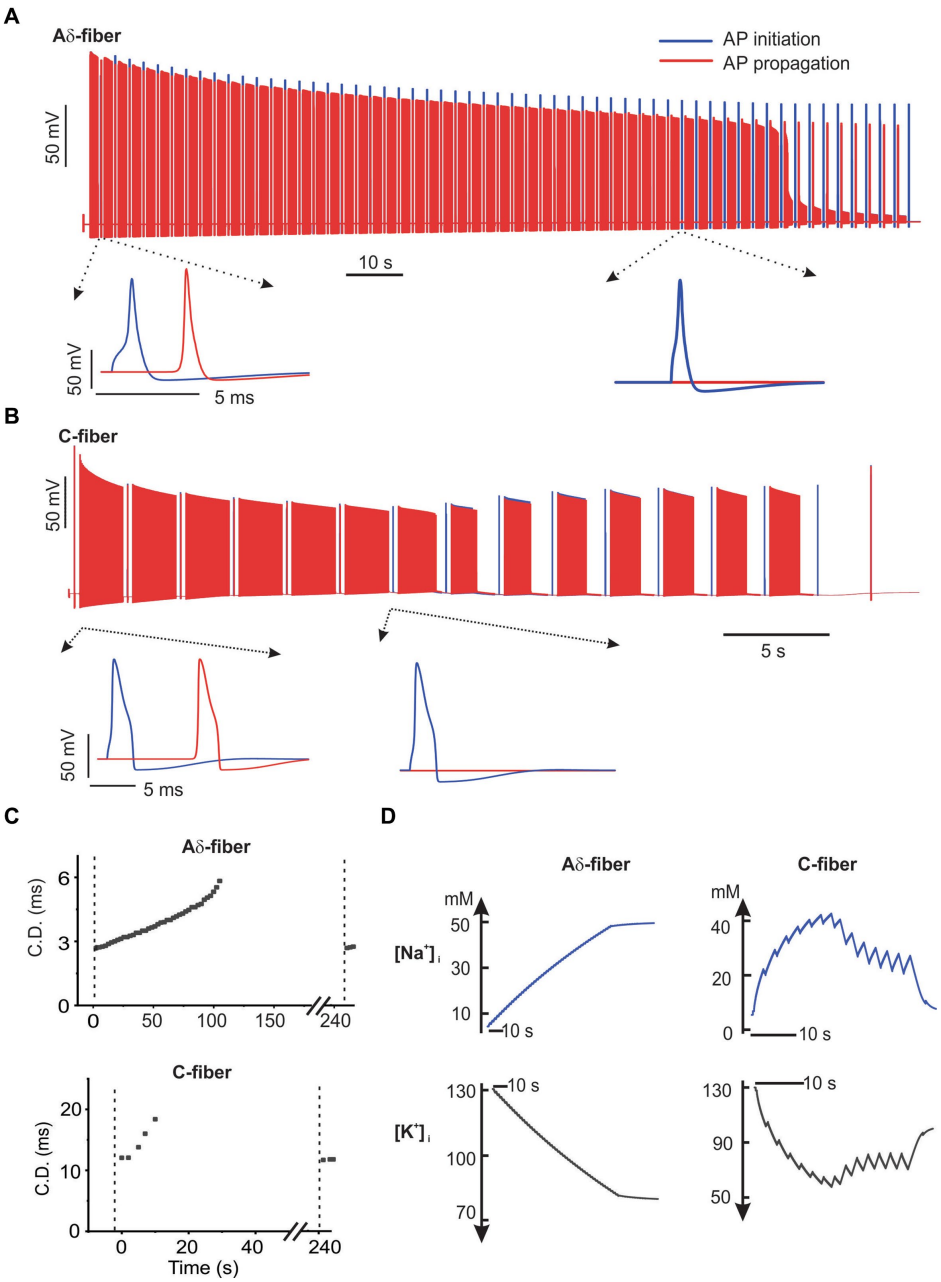
The NEURON simulation of axonal transmission block by ePNS. The model-simulated transmembrane potential voltages from the onset of ePNS till conduction block was plotted in **(A)** for the Aδ-fiber model and in **(B)** for the C-fiber model. **(C)** The model-simulated conduction delay (CD) following the ePNS protocol. The CD was calculated once every 2.5 s. **(D)** The model-simulated change in intracellular Na^+^ and K^+^ concentrations during the ePNS protocol.

To evaluate the influence of neuromodulation on afferent neural transmission, we employed a similar synchronized stimulation protocol as outlined in Figure 1B, including trains of 2-s-long ePNS separated by 0.5-s-long intertrain intervals with no stimulation. Biphasic ePNS (with a negative phase first) was delivered at 20 Hz in the C-fiber model (0.5 mA amplitude, 0.5 ms duration) and at 100 Hz in the Aδ-fiber model (0.5 mA amplitude, 0.5 ms duration). In the Aδ-fiber model, the model-simulated transmembrane potential voltages at both ends of the axon, i.e., the AP initiation and propagation sites, were plotted in Figure 7A from the onset of ePNS until conduction block. In the extended view, it is apparent that AP transmission to the distal end occurred before the ePNS and was completely blocked approximately 105 s after the onset of the 100-Hz ePNS. Similarly, as shown in Figure 7B, the transmembrane potential voltages in the C-fiber model showed complete transmission block approximately 20 s after the onset of the 20-Hz ePNS. In correspondence with the experimental data on CD following the ePNS protocol (Figure 4), displayed in Figure 7C are the model-simulated increases in CD in both the Aδ-fiber and C-fiber models, showing a CDI_max_ of ~80% for both. It is worth noting that conduction resumes approximately 60 s after the stimulation is terminated. Displayed in Figure 7D are intracellular Na^+^ ([Na^+^]_i_) and K^+^ concentrations ([K^+^]_i_) following the ePNS protocol, which change significantly following each 2-s train of stimulation. Initially, the uptick in [Na^+^]_i_ per stimulus train was approximately 2.5 mM for Aδ-fibers and 25 mM for C-fibers. This rise in [Na^+^]_i_ gradually reached a plateau, stabilizing at around 50 mM for Aδ-fibers and 25 mM for C-fibers. Similarly, the decline in intracellular K^+^ concentration ([K^+^]_i_) amounted to approximately 2.5 mM per stimulus train in Aδ-fibers and 25 mM in C-fibers, eventually stabilizing at 80 mM and 60 mM, respectively.

**Figure 8:**
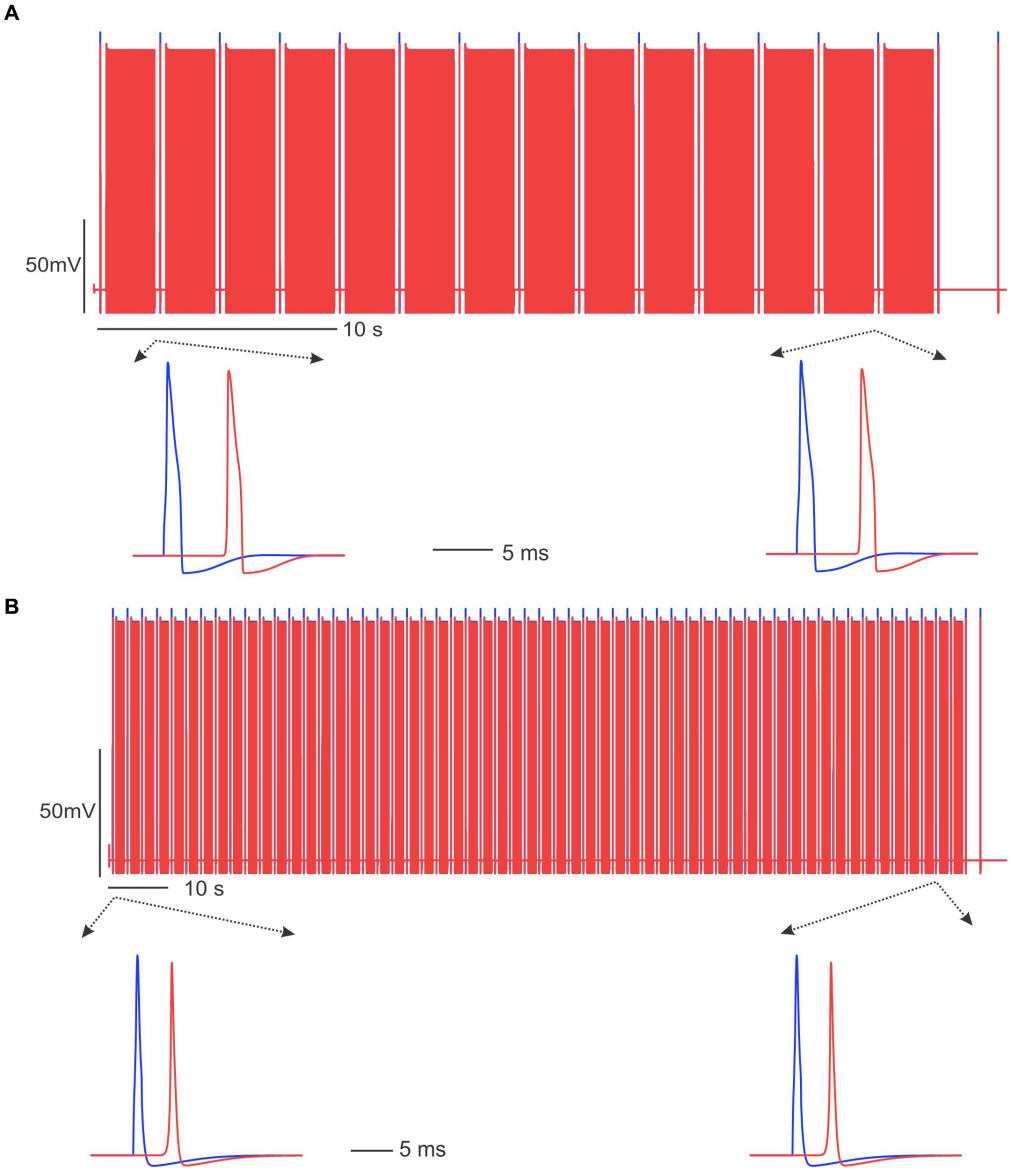
Sub-kilohertz stimulation failed to block the AP transmission in the absence of disrupted transmembrane ionic gradients. **(A)** Stimulation at 20 Hz did not block the C-fiber model. **(B)** The Aδ-fiber model was not blocked by 50 Hz stimulation.

To evaluate the essential role of disrupted transmembrane ionic gradients in conduction block, we maintained constant intra-axonal and extra-axonal ionic concentrations throughout the simulation. As demonstrated in Figure 8, under these conditions, 20 Hz and 50 Hz ePNS failed to block the AP transmission in the C-fiber and Aδ-fiber models, respectively. These results suggest that the disruption of transmembrane ionic gradients is a necessary factor for the induction of conduction block by sub-kilohertz stimulation in our models.

## 4 Discussion

In mammalian peripheral nerves, the transmission of axonal action potentials (APs) in large, myelinated axons is robust, enabling sustained transmission of trains of impulses at 100 Hz or higher for hours (Prochazka and Gorassini, 1998). Consequently, electrical pulse stimulation to block the conduction of A-fiber axons typically requires the stimulus frequency in the kilohertz range, often exceeding 10 kHz (Kilgore and Bhadra, 2014; Patel and Butera, 2018). The frequency range effective for blocking A-fiber transmission has been established through either direct recordings of compound action potentials (CAP) from a population of A-fiber axons in whole-nerve configurations (Juan et al., 2014) or indirect measurement of evoked forces in muscles innervated by motor A-fiber axons (Dowden et al., 2010). The muscle force measurement is not applicable for studying unmyelinated C-fiber axons or thinly myelinated Aδ-fiber axons, as they do not directly influence muscle contraction. CAP recordings from unmyelinated C-fibers pose challenges due to their small signal amplitudes, limiting resolution for assessing subtle neuromodulatory effects. Unlike the monopole-like transmembrane currents from saltatory transmission in myelinated A-fiber axons, AP transmission in slow-conducting C-fibers generates dipole-like transmembrane currents that resulted in a significantly lower extracellular electrical field compared to the A-fiber currents (Plonsey and Barr, 2007). Thus, extracellular recordings of action potentials from Aδ- or C-fiber axons require close proximity of the recording electrode to the nerve axon, typically achieved by manually teasing nerve bundles into fine filaments approximately 10 microns thick (e.g., Chen et al., 2017a; Ilham et al., 2018; Liu et al., 2021). Recent studies by us and others implemented this teased fiber approach to record from split dorsal roots of rats (Chao et al., 2020) and mice (Chen et al., 2022), respectively. Both studies reported reversible blocking of AP transmission in Aδ- and C-fiber afferents by sub-kilohertz pulse stimulation of the dorsal root ganglion (DRG). Especially, frequency as low as 10 Hz is capable of blocking the transmission in C-fiber afferents.

In the current study, we focused on peripheral nerve trunks and demonstrated that ePNS from outside the epineurium can achieve similar transmission block of Aδ- and C-fiber axons in three mouse peripheral nerves: two spinal nerves and one autonomic nerve. The nerve blocking effect by sub-kilohertz ePNS aligns with our prior DRG stimulation study in three aspects (Chen et al., 2022). First, both DRG stimulation and ePNS require suprathreshold stimulation for nerve transmission block, as subthreshold stimulation without evoked action potentials had no significant effect on nerve conduction delay. Second, the range of stimulus frequency efficiently blocking conduction depends on axonal size, with the optimal blocking frequency (OBF) ranging from 10 to 50 Hz for unmyelinated C-fibers and 50–1,000 Hz for myelinated Aδ-fibers. Third, similar increases in conduction delay follow the onset of both ePNS and DRG stimulation until complete conduction block. The DRG was originally hypothesized as the block location due to unique anatomical structures of afferent neurons there, including stem axons, T-junctions, and somata. However, the exclusion of DRG and dorsal roots in the current nerve blocking study strongly indicates the nerve axons as the location of conduction block. In support, the OBF for Aδ-fibers is comparable between DRG stimulation and ePNS, and C-fibers are efficiently blocked by 10–50 Hz stimulation in both cases. It is worth noting that DRG stimulation at 100–1,000 Hz blocked over 50% of C-fiber afferents (Chen et al., 2022), whereas in current study ePNS in the same frequency range blocked no more than 5% of C-fiber axons. This suggests the differential excitability of C-fiber afferents to high frequency stimulation (100–1,000 Hz) at the spinal nerve versus DRG. Indeed, a needle electrode placed at epidural DRG was sufficient to evoke C-fiber afferent activities whereas a glass suction electrode was required to efficiently excite C-fiber axons protected by multiple connective tissue layers within the peripheral nerve trunk. It is speculated that the connective tissues within the nerve trunk may act as a low-pass filter, dampening the intensity of high-frequency stimulation. This could lead to inadequate activation of C-fiber axons with stimulation frequencies ranging from 100 to 1,000 Hz, potentially resulting in the absence of a conduction block.

The finding that stimulation as low as 10 Hz can block afferent transmission has significant clinical implications for pain management with peripheral neuromodulation. The conventional “Gate Control” theory first published by Melzack and Wall (1965) suggests that neurostimulators relieve pain by activating low-threshold, myelinated afferents, triggering paresthesia, a non-painful tingling sensation that masks nociceptive signals (Shamji et al., 2017). However, many patients receiving DRG stimulation do not require paresthesia to achieve pain relief; paresthesia-free patients reported comparable and even better therapeutical benefit from DRG stimulation as patients experiencing paresthesia (Verrills et al., 2019; Mekhail et al., 2020). Thus, it is possible that nociceptor signaling from unmyelinated C-fiber afferents can be blocked by DRG stimulation with clinically applied stimulus intensities, which activates the C-fiber afferents at the OBF to cause conduction block. In contrast, the ePNS has been reported as the validation of the “Gate Control Theory,” and paresthesia appears necessary for its pain-relieving effect (Weiner, 2003). This indicates that most C-fiber afferents are not activated by ePNS at clinically applied intensities. Both clinical observations and preclinical experimental studies indicate that C-fibers are more difficult to evoke by peripheral nerve stimulation than by DRG stimulation. Further research is warranted to investigate underlying anatomical and functional characteristics accounting for this threshold difference in evoking C-fiber afferents at the DRG versus the nerve trunk.

Both Aδ- and C-fiber afferents exhibit pronounced activity-dependent conduction slowing when stimulated at their respective OBF. This phenomenon has been observed in microneurographic studies on human peripheral afferents, which demonstrated progressive slowing in C-fibers using a 2-Hz stimulation protocol (Serra et al., 1999). A complementary computational modeling study suggests that the gradual increase in intra-axonal sodium concentration contributes to this conduction slowing (Tigerholm et al., 2013). With a 2-Hz stimulation, the increase in conduction delay (CD) typically plateaus at about 10% and is usually no more than 30% (Schmelz et al., 2000; Schmidt et al., 2002; Schmelz and Schmidt, 2009; Obreja et al., 2010). However, nerve conduction block was not assessed or reported in those microneurographic studies. In current study, we monitored CD every 2 s during the ePNS protocol that stimulates at frequencies much higher than 2 Hz, revealing a progressive increase in CD until conduction block. The maximum increase in CD (CDI_max_) varies greatly between samples, reaching levels as high as ~100%. Furthermore, the notably higher level of CDI_max_ in blocked axons compared to unblocked ones suggests that the extent to which conduction delay can be significantly increased by a particular ePNS protocol may serve as a predictor of the likelihood of conduction block in the stimulated axon. It is worth noting that the *ex vivo* setting used in our current study may not fully represent the complex homeostatic mechanisms present in an *in vivo* context. These differences could potentially lead to divergent outcomes in terms of the effectiveness and duration of the conduction block. We acknowledge that the findings from our *ex vivo* study will require further validation through carefully designed *in vivo* experiments to confirm their translational relevance.

Our computational simulation of action potential initiation and propagation reproduces the experimental results of conduction block in both Aδ- and C-fiber axons, capturing the progressive increase in conduction delay (CD) following ePNS until transmission block. We heavily utilized components from our prior computational model of a mouse colorectal afferent, including various subtypes of sodium and potassium ion channels, the sodium, potassium-ATPase (NaKA), and the tracking of intra-axonal ionic concentration (Feng et al., 2015). Specifically, we simulated variations in intracellular Na^+^ and K^+^ concentrations resulting from transmembrane ionic current flow and axial ionic diffusion, showing significant changes following a 2-s stimulation train at 20 Hz for the C-fiber model and at 100 Hz for the A-fiber model. In the C-fiber model with a 1-micron diameter, Na^+^ and K^+^ concentrations exhibited a millimolar change per action potential. This aligns with a simple calculation assuming axonal membrane capacitance charging exclusively through transmembrane Na^+^ current and discharging exclusively through K^+^ current. Our modeling simulations strongly suggest that disruption of transmembrane Na^+^ and K^+^ concentration gradients underly the transmission block by ePNS. Notably, our model predicts a reduction in the sum of intracellular Na^+^ and K^+^ concentration following ePNS, caused by a net outward ionic flow through the NaKA due to the 3: 2 transport ratio of Na^+^ and K^+^ ions. This model-predicted reduction in intra-axonal osmolarity is supported by experimental observations from myelinated A-fiber axons undergoing prolonged 100 Hz high-frequency stimulation, which demonstrated a significant increase in the periaxonal space due to osmotically driven water diffusion (Trigo and Smith, 2015).

The current computational model provides one plausible mechanism of conduction block, i.e., the dysregulated transmembrane ionic gradients. When the extra- and intra-axonal ionic concentrations are kept constant, the same neuromodulation protocols failed to block the transmission in C-fiber and Aδ-fiber models (Figure 8). However, it is important to acknowledge several limitations of the current model, which potentially excludes the interrogation of other mechanisms that may contribute to conduction block. The discrepancy of the longer duration required for block in the model simulation than in the experimental observation clearly indicates the involvement of additional mechanisms in the transmission block process. First, our current model does not consider ionic and metabotropic mechanisms mediated by calcium ions. There is a significant increase in calcium concentration in peripheral axons following action potential conduction (Anderson et al., 2018), which likely play a modulatory role in AP conduction velocity and slowing. Future experimental and computational studies should focus on the role of calcium in stimulation-induced conduction block. Second, the potential reduction in intra-axonal resistivity due to the altered Na^+^ and K^+^ concentrations is not accounted for in the current model. This reduction could potentially play a role in expediting the conduction block by ePNS. Third, the model parameters for the C-fiber and Aδ-fiber are tuned to encode at low frequencies (no more than 50 Hz). Thus, the current model is not suitable to assess the blocking effect of high-frequency stimulation above 100 Hz, as it will not allow us to tease out the intrinsic model properties from other effects that account for the failed block by high-frequency stimulation. Finally, the current gating kinetics of the Na^+^ and K^+^ channels do not include time constants on the order of minutes, which is the time scale for recovery from conduction block. New equations for the voltage-gated ion channels are required for a focused study on the role of extra-slow channel inactivation on the scale of minutes in the transmission block.

## 5 Conclusion

The current study presents direct experimental evidence unveiling the reversible conduction block of Aδ- and C-fiber peripheral axons by ePNS within the sub-kilohertz range. More than 88% of Aδ- and C-fibers in three distinct peripheral nerves were blocked by at least one of the five stimulus frequencies tested: 10, 50, 100, 500, and 1,000 Hz. Conversely, no axon was blocked by 5-Hz stimulation. Notably, the efficacy of ePNS-induced blockage depends on the stimulation frequencies relative to the conduction velocities of the targeted nerve axons; slow-conducting C-fibers are optimally blocked by 10–50 Hz stimulation, while Aδ-fibers are effectively blocked by 100–1,000 Hz stimulation. ePNS leads to a progressive increase in conduction delay until transmission blockage, with blocked axons exhibiting significantly higher maximum conduction delay increase than unblocked ones. Additionally, complementary computational modeling of action potential transmission mirrors the blocking effects observed with extracellular pulse stimulation, indicating that disrupted trans-axonal ionic concentration gradients contribute to ePNS-induced conduction block. These findings offer a novel nerve-blocking mechanism that could be leveraged by peripheral neuromodulation methods to enhance therapeutic interventions for managing chronic pain and other neurological disorders.

## Data availability statement

The raw data supporting the conclusions of this article will be made available by the authors, without undue reservation.

## Ethics statement

The animal study was approved by University of Connecticut Institutional Animal Care and Use Committee. The study was conducted in accordance with the local legislation and institutional requirements.

## Author contributions

SZ: Data curation, Methodology, Writing – original draft, Writing – review & editing, Formal analysis, Investigation. LC: Investigation, Methodology, Writing – review & editing. SL: Investigation, Methodology, Writing – review & editing, Formal analysis, Writing – original draft. AS: Investigation, Methodology, Writing – review & editing. JL: Investigation, Methodology, Writing – review & editing, Data curation. BF: Data curation, Methodology, Writing – review & editing, Conceptualization, Funding acquisition, Project administration, Resources, Supervision, Validation, Visualization, Writing – original draft.
